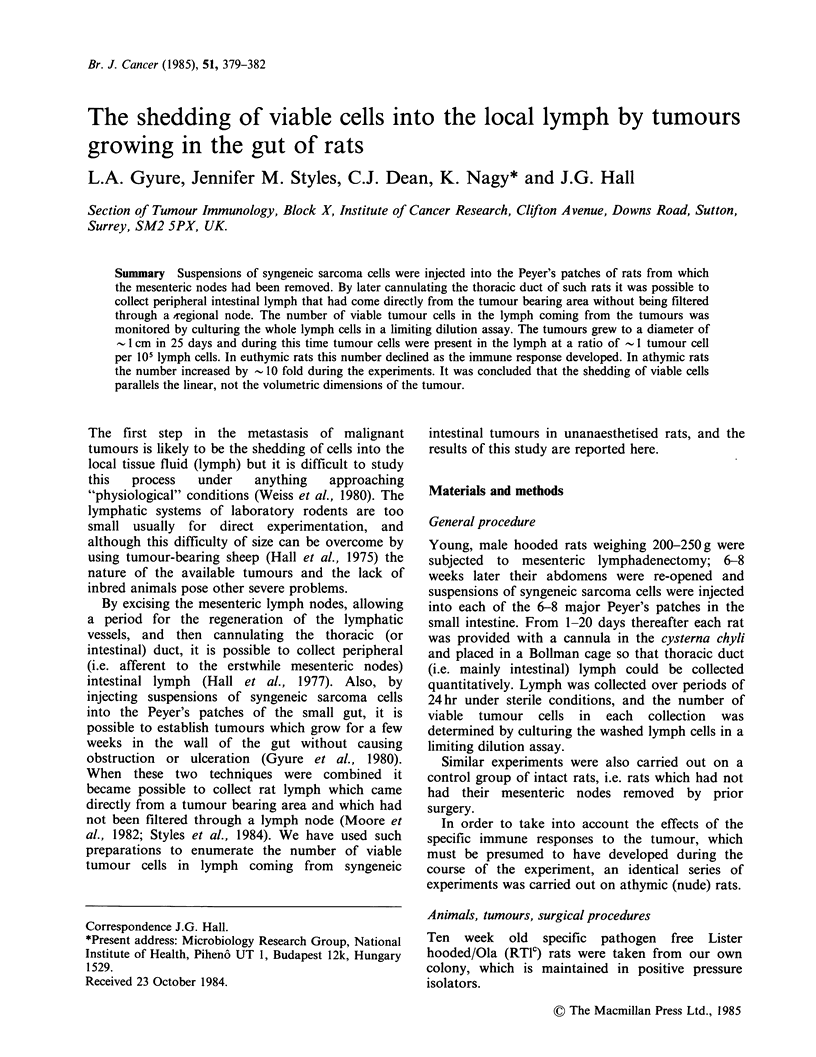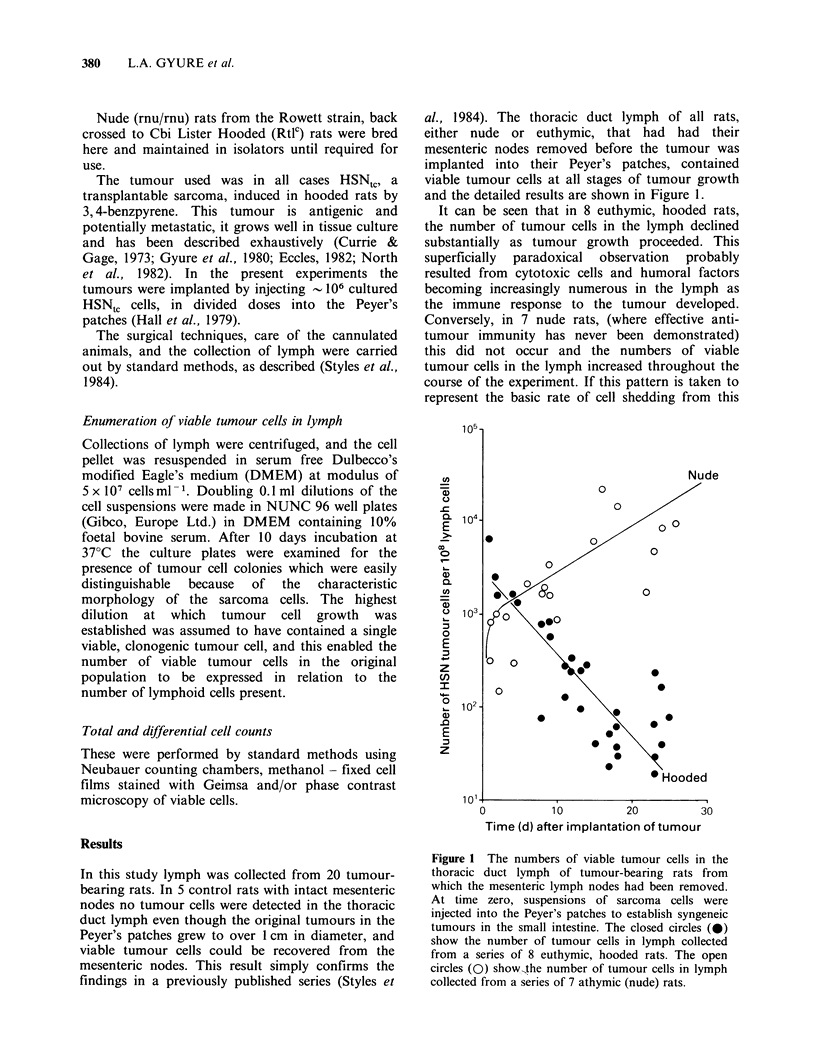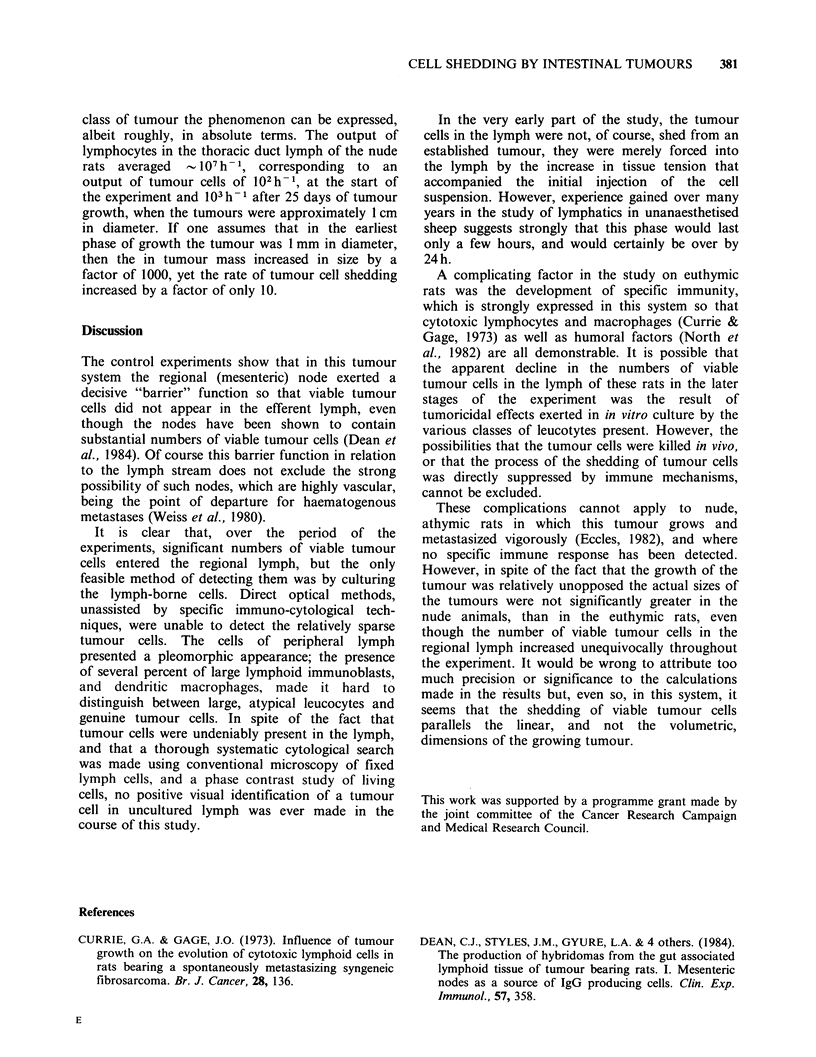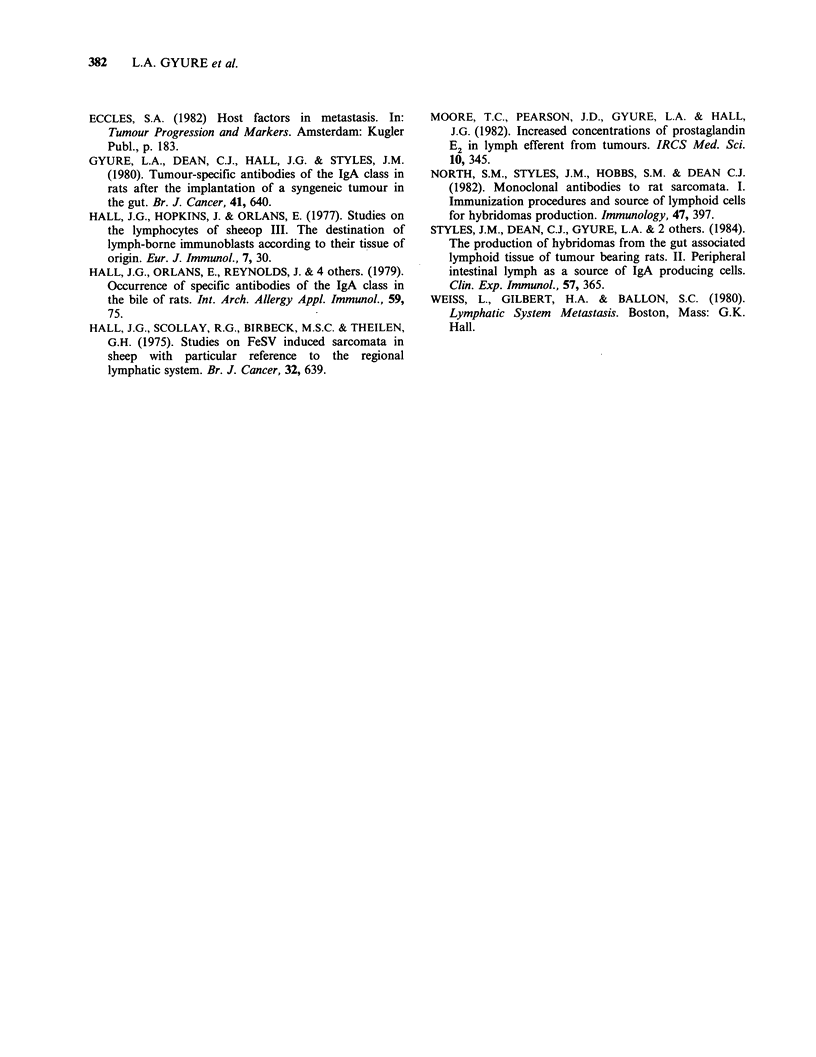# The shedding of viable cells into the local lymph by tumours growing in the gut of rats.

**DOI:** 10.1038/bjc.1985.50

**Published:** 1985-03

**Authors:** L. A. Gyure, J. M. Styles, C. J. Dean, K. Nagy, J. G. Hall

## Abstract

Suspensions of syngeneic sarcoma cells were injected into the Peyer's patches of rats from which the mesenteric nodes had been removed. By later cannulating the thoracic duct of such rats it was possible to collect peripheral intestinal lymph that had come directly from the tumour bearing area without being filtered through a regional node. The number of viable tumour cells in the lymph coming from the tumours was monitored by culturing the whole lymph cells in a limiting dilution assay. The tumours grew to a diameter of approximately 1 cm in 25 days and during this time tumour cells were present in the lymph at a ratio of approximately 1 tumour cell per 10(5) lymph cells. In euthymic rats this number declined as the immune response developed. In athymic rats the number increased by approximately 10 fold during the experiments. It was concluded that the shedding of viable cells parallels the linear, not the volumetric dimensions of the tumour.


*Br. J. Cancer* (1985), **51**, 379–382

### The shedding of viable cells into the local lymph by tumours growing in the gut of rats

L.A. Gyure, Jennifer M. Styles, C.J. Dean, K. Nagy* and J.G. Hall

*Section of Tumour Immunology, Block X, Institute of Cancer Research, Clifton Avenue, Downs Road, Sutton, Surrey, SM2 5PX, UK.*

**Summary** Suspensions of syngeneic sarcoma cells were injected into the Peyer's patches of rats from which the mesenteric nodes had been removed. By later cannulating the thoracic duct of such rats it was possible to collect peripheral intestinal lymph that had come directly from the tumour bearing area without being filtered through a regional node. The number of viable tumour cells in the lymph coming from the tumours was monitored by culturing the whole lymph cells in a limiting dilution assay. The tumours grew to a diameter of ~1 cm in 25 days and during this time tumour cells were present in the lymph at a ratio of ~1 tumour cell per $10^5$ lymph cells. In euthymic rats this number declined as the immune response developed. In athymic rats the number increased by ~10 fold during the experiments. It was concluded that the shedding of viable cells parallels the linear, not the volumetric dimensions of the tumour.

The first step in the metastasis of malignant tumours is likely to be the shedding of cells into the local tissue fluid (lymph) but it is difficult to study this process under anything approaching "physiological" conditions (Weiss *et al.*, 1980). The lymphatic systems of laboratory rodents are too small usually for direct experimentation, and although this difficulty of size can be overcome by using tumour-bearing sheep (Hall *et al.*, 1975) the nature of the available tumours and the lack of inbred animals pose other severe problems.

By excising the mesenteric lymph nodes, allowing a period for the regeneration of the lymphatic vessels, and then cannulating the thoracic (or intestinal) duct, it is possible to collect peripheral (i.e. afferent to the erstwhile mesenteric nodes) intestinal lymph (Hall *et al.*, 1977). Also, by injecting suspensions of syngeneic sarcoma cells into the Peyer's patches of the small gut, it is possible to establish tumours which grow for a few weeks in the wall of the gut without causing obstruction or ulceration (Gyure *et al.*, 1980). When these two techniques were combined it became possible to collect rat lymph which came directly from a tumour bearing area and which had not been filtered through a lymph node (Moore *et al.*, 1982; Styles *et al.*, 1984). We have used such preparations to enumerate the number of viable tumour cells in lymph coming from syngeneic intestinal tumours in unanaesthetised rats, and the results of this study are reported here.

Correspondence J.G. Hall.
*Present address: Microbiology Research Group, National Institute of Health, Pihenô UT 1, Budapest 12k, Hungary 1529.
Received 23 October 1984.

### Materials and methods

### *General procedure*

Young, male hooded rats weighing 200–250 g were subjected to mesenteric lymphadenectomy; 6–8 weeks later their abdomens were re-opened and suspensions of syngeneic sarcoma cells were injected into each of the 6–8 major Peyer's patches in the small intestine. From 1–20 days thereafter each rat was provided with a cannula in the *cysterna chyli* and placed in a Bollman cage so that thoracic duct (i.e. mainly intestinal) lymph could be collected quantitatively. Lymph was collected over periods of 24 hr under sterile conditions, and the number of viable tumour cells in each collection was determined by culturing the washed lymph cells in a limiting dilution assay.

Similar experiments were also carried out on a control group of intact rats, i.e. rats which had not had their mesenteric nodes removed by prior surgery.

In order to take into account the effects of the specific immune responses to the tumour, which must be presumed to have developed during the course of the experiment, an identical series of experiments was carried out on athymic (nude) rats.

### *Animals, tumours, surgical procedures*

Ten week old specific pathogen free Lister hooded/Ola ($RT1^c$) rats were taken from our own colony, which is maintained in positive pressure isolators.

© The Macmillan Press Ltd., 1985

Nude (rnu/rnu) rats from the Rowett strain, back crossed to Cbi Lister Hooded (Rtl$^c$) rats were bred here and maintained in isolators until required for use.

The tumour used was in all cases $HSN_{tc}$, a transplantable sarcoma, induced in hooded rats by 3,4-benzpyrene. This tumour is antigenic and potentially metastatic, it grows well in tissue culture and has been described exhaustively (Currie & Gage, 1973; Gyure *et al.*, 1980; Eccles, 1982; North *et al.*, 1982). In the present experiments the tumours were implanted by injecting $\sim 10^6$ cultured $HSN_{tc}$ cells, in divided doses into the Peyer's patches (Hall *et al.*, 1979).

The surgical techniques, care of the cannulated animals, and the collection of lymph were carried out by standard methods, as described (Styles *et al.*, 1984).

*Enumeration of viable tumour cells in lymph*

Collections of lymph were centrifuged, and the cell pellet was resuspended in serum free Dulbecco's modified Eagle's medium (DMEM) at modulus of $5 \times 10^7$ cells $ml^{-1}$. Doubling 0.1 ml dilutions of the cell suspensions were made in NUNC 96 well plates (Gibco, Europe Ltd.) in DMEM containing 10% foetal bovine serum. After 10 days incubation at 37°C the culture plates were examined for the presence of tumour cell colonies which were easily distinguishable because of the characteristic morphology of the sarcoma cells. The highest dilution at which tumour cell growth was established was assumed to have contained a single viable, clonogenic tumour cell, and this enabled the number of viable tumour cells in the original population to be expressed in relation to the number of lymphoid cells present.

*Total and differential cell counts*

These were performed by standard methods using Neubauer counting chambers, methanol – fixed cell films stained with Geimsa and/or phase contrast microscopy of viable cells.

### Results

In this study lymph was collected from 20 tumour-bearing rats. In 5 control rats with intact mesenteric nodes no tumour cells were detected in the thoracic duct lymph even though the original tumours in the Peyer's patches grew to over 1 cm in diameter, and viable tumour cells could be recovered from the mesenteric nodes. This result simply confirms the findings in a previously published series (Styles *et al.*, 1984). The thoracic duct lymph of all rats, either nude or euthymic, that had had their mesenteric nodes removed before the tumour was implanted into their Peyer's patches, contained viable tumour cells at all stages of tumour growth and the detailed results are shown in Figure 1.

It can be seen that in 8 euthymic, hooded rats, the number of tumour cells in the lymph declined substantially as tumour growth proceeded. This superficially paradoxical observation probably resulted from cytotoxic cells and humoral factors becoming increasingly numerous in the lymph as the immune response to the tumour developed. Conversely, in 7 nude rats, (where effective anti-tumour immunity has never been demonstrated) this did not occur and the numbers of viable tumour cells in the lymph increased throughout the course of the experiment. If this pattern is taken to represent the basic rate of cell shedding from this

**Figure 1** The numbers of viable tumour cells in the thoracic duct lymph of tumour-bearing rats from which the mesenteric lymph nodes had been removed. At time zero, suspensions of sarcoma cells were injected into the Peyer's patches to establish syngeneic tumours in the small intestine. The closed circles (●) show the number of tumour cells in lymph collected from a series of 8 euthymic, hooded rats. The open circles (○) show the number of tumour cells in lymph collected from a series of 7 athymic (nude) rats.

class of tumour the phenomenon can be expressed, albeit roughly, in absolute terms. The output of lymphocytes in the thoracic duct lymph of the nude rats averaged $\sim 10^7 h^{-1}$, corresponding to an output of tumour cells of $10^2 h^{-1}$, at the start of the experiment and $10^3 h^{-1}$ after 25 days of tumour growth, when the tumours were approximately 1 cm in diameter. If one assumes that in the earliest phase of growth the tumour was 1 mm in diameter, then the in tumour mass increased in size by a factor of 1000, yet the rate of tumour cell shedding increased by a factor of only 10.

### Discussion

The control experiments show that in this tumour system the regional (mesenteric) node exerted a decisive "barrier" function so that viable tumour cells did not appear in the efferent lymph, even though the nodes have been shown to contain substantial numbers of viable tumour cells (Dean *et al.*, 1984). Of course this barrier function in relation to the lymph stream does not exclude the strong possibility of such nodes, which are highly vascular, being the point of departure for haematogenous metastases (Weiss *et al.*, 1980).

It is clear that, over the period of the experiments, significant numbers of viable tumour cells entered the regional lymph, but the only feasible method of detecting them was by culturing the lymph-borne cells. Direct optical methods, unassisted by specific immuno-cytological techniques, were unable to detect the relatively sparse tumour cells. The cells of peripheral lymph presented a pleomorphic appearance; the presence of several percent of large lymphoid immunoblasts, and dendritic macrophages, made it hard to distinguish between large, atypical leucocytes and genuine tumour cells. In spite of the fact that tumour cells were undeniably present in the lymph, and that a thorough systematic cytological search was made using conventional microscopy of fixed lymph cells, and a phase contrast study of living cells, no positive visual identification of a tumour cell in uncultured lymph was ever made in the course of this study.

In the very early part of the study, the tumour cells in the lymph were not, of course, shed from an established tumour, they were merely forced into the lymph by the increase in tissue tension that accompanied the initial injection of the cell suspension. However, experience gained over many years in the study of lymphatics in unanaesthetised sheep suggests strongly that this phase would last only a few hours, and would certainly be over by 24 h.

A complicating factor in the study on euthymic rats was the development of specific immunity, which is strongly expressed in this system so that cytotoxic lymphocytes and macrophages (Currie & Gage, 1973) as well as humoral factors (North *et al.*, 1982) are all demonstrable. It is possible that the apparent decline in the numbers of viable tumour cells in the lymph of these rats in the later stages of the experiment was the result of tumoricidal effects exerted in *in vitro* culture by the various classes of leucotytes present. However, the possibilities that the tumour cells were killed *in vivo*, or that the process of the shedding of tumour cells was directly suppressed by immune mechanisms, cannot be excluded.

These complications cannot apply to nude, athymic rats in which this tumour grows and metastasized vigorously (Eccles, 1982), and where no specific immune response has been detected. However, in spite of the fact that the growth of the tumour was relatively unopposed the actual sizes of the tumours were not significantly greater in the nude animals, than in the euthymic rats, even though the number of viable tumour cells in the regional lymph increased unequivocally throughout the experiment. It would be wrong to attribute too much precision or significance to the calculations made in the results but, even so, in this system, it seems that the shedding of viable tumour cells parallels the linear, and not the volumetric, dimensions of the growing tumour.

This work was supported by a programme grant made by the joint committee of the Cancer Research Campaign and Medical Research Council.

### References

CURRIE, G.A. & GAGE, J.O. (1973). Influence of tumour growth on the evolution of cytotoxic lymphoid cells in rats bearing a spontaneously metastasizing syngeneic fibrosarcoma. *Br. J. Cancer*, **28**, 136.

DEAN, C.J., STYLES, J.M., GYURE, L.A. & 4 others. (1984). The production of hybridomas from the gut associated lymphoid tissue of tumour bearing rats. I. Mesenteric nodes as a source of IgG producing cells. *Clin. Exp. Immunol.*, **57**, 358.

ECCLES, S.A. (1982) Host factors in metastasis. In: *Tumour Progression and Markers*. Amsterdam: Kugler Publ., p. 183.

GYURE, L.A., DEAN, C.J., HALL, J.G. & STYLES, J.M. (1980). Tumour-specific antibodies of the IgA class in rats after the implantation of a syngeneic tumour in the gut. *Br. J. Cancer*, **41**, 640.

HALL, J.G., HOPKINS, J. & ORLANS, E. (1977). Studies on the lymphocytes of sheeop III. The destination of lymph-borne immunoblasts according to their tissue of origin. *Eur. J. Immunol.*, **7**, 30.

HALL, J.G., ORLANS, E., REYNOLDS, J. & 4 others. (1979). Occurrence of specific antibodies of the IgA class in the bile of rats. *Int. Arch. Allergy Appl. Immunol.*, **59**, 75.

HALL, J.G., SCOLLAY, R.G., BIRBECK, M.S.C. & THEILEN, G.H. (1975). Studies on FeSV induced sarcomata in sheep with particular reference to the regional lymphatic system. *Br. J. Cancer*, **32**, 639.

MOORE, T.C., PEARSON, J.D., GYURE, L.A. & HALL, J.G. (1982). Increased concentrations of prostaglandin $E_2$ in lymph efferent from tumours. *IRCS Med. Sci.* **10**, 345.

NORTH, S.M., STYLES, J.M., HOBBS, S.M. & DEAN C.J. (1982). Monoclonal antibodies to rat sarcomata. I. Immunization procedures and source of lymphoid cells for hybridomas production. *Immunology*, **47**, 397.

STYLES, J.M., DEAN, C.J., GYURE, L.A. & 2 others. (1984). The production of hybridomas from the gut associated lymphoid tissue of tumour bearing rats. II. Peripheral intestinal lymph as a source of IgA producing cells. *Clin. Exp. Immunol.*, **57**, 365.

WEISS, L., GILBERT, H.A. & BALLON, S.C. (1980). *Lymphatic System Metastasis*. Boston, Mass: G.K. Hall.